# Asian sand dust aggregate causes atopic dermatitis-like symptoms in Nc/Nga mice

**DOI:** 10.1186/s13223-015-0068-y

**Published:** 2015-01-23

**Authors:** Sayaka Takeshita, Takahiro Tokunaga, Yoshiko Tanabe, Tadao Arinami, Takamichi Ichinose, Emiko Noguchi

**Affiliations:** Department of Medical Genetics, Faculty of Medicine, University of Tsukuba, 1-1-1 Tennoudai, Tsukuba, Ibaraki-ken 305-8575 Japan; Department of Otorhinolaryngology Head and Neck Surgery, Faculty of Medical Sciences, University of Fukui, Fukui, Japan; Department of Health Sciences, Oita University of Nursing and Health Sciences, Notsuharu, Oita Japan; Japan Science and Technology Agency, Core Research for Evolutional Science and Technology (CREST), Chiyoda-ku, Tokyo Japan

**Keywords:** Dermatitis, Atopic, Dermatophagoides farina, Mice

## Abstract

**Background:**

Asian sand dust (ASD) originates from the arid and semiarid areas of China, and epidemiologic studies have shown that ASD exposure is associated with various allergic and respiratory symptoms. However, few studies have been performed to assess the relationship between skin inflammation and ASD exposure.

**Methods:**

Twelve-week-old NC/Nga mice were divided into 6 groups (n = 8 for each group): hydrophilic petrolatum only (control); hydrophilic petrolatum plus ASD (ASD); hydrophilic petrolatum and heat inactivated-ASD (H-ASD); *Dermatophagoides farinae* extract (Df); Df and ASD (Df + ASD), and; Df and H-ASD (Df + H-ASD). The NC/Nga mice in each group were subjected to treatment twice a week for 4 weeks. We evaluated skin lesions by symptoms, pathologic changes, and serum IgE levels.

**Results:**

ASD alone did not induce atopic dermatitis (AD)-like skin symptoms. However, Df alone, Df + H-ASD and Df + ASD all induced AD-like symptoms, and dermatitis scores in the group of Df + ASD group were significantly greater than that of the Df group (P = 0.0011 at day 21; and P = 0.017 at day 28). Mean serum IgE was markedly increased in the Df and Df + ASD groups, compared to the ASD and control groups (P < 0.0001), and serum IgE levels in the Df + ASD group were significantly higher compared to the Df group (P = 0.003).

**Conclusions:**

ASD alone did not cause AD-like symptoms in NC/Nga mice. However, AD-like symptoms induced by Df, a major allergen, were enhanced by adding ASD. Although no epidemiological studies have been conducted for the association between ASD and symptoms of dermatitis, our data suggest that it is likely that ASD may contribute to the exacerbation of not only respiratory symptoms, but also skin diseases, in susceptible individuals.

## Background

Atopic dermatitis (AD) is a hereditary, pruritic, inflammatory, chronic skin disease that occurs most commonly in early childhood, with an increasing prevalence observed especially in industrial countries [1]. The histology of AD is characterized by epidermal alterations, and perivascular infiltrate of lymphocytes, monocytes, macrophages, dendritic cells, and a few eosinophils in the dermis [2]. AD often shows a disturbance of epidermal-barrier function that culminates in dry skin, and immunoglobulin (Ig)E-mediated sensitization to food and environmental allergens are often observed in patients with AD [3].

Various factors, including both immunological and non-immunological abnormalities, contribute to the pathogenesis and development of AD. These factors are not completely understood, but inflammatory immune dysregulation that causes IgE-mediated sensitization, and skin barrier dysfunctions, are likely to play important roles in the development of AD.

Asian sand dust (ASD) originates from the arid and semiarid areas of China (the Gobi Desert and the Ocher Plateau), and is transported from East Asia to countries in the Pacific region, including South Korea, Japan, and sometimes as far as to the United States, during the spring season. ASD contains various chemicals, such as sulfate or nitrate derived from alkaline soil, as well as microbiological materials [4]. Silica, a major mineralogical component of Asian sand dust, has been linked to pulmonary diseases, including tuberculosis, chronic bronchitis, and lung cancer [5]. Epidemiologic studies have shown that ASD exposure is associated with asthma hospitalization in children [6], ASD-events, and pneumonia admissions [7]. Animal experiments have also demonstrated that ASD can enhance murine lung inflammation by pathogens [8] and allergens [9,10]. However, few studies have been performed to investigate the relationship between inflammation of the skin, and ASD exposure.

NC/Nga mice were established in 1957 as an inbred strain based on Japanese fancy mice [11], and this strain spontaneously develops dermatitis associated with excessive IgE production when animals are raised under conventional conditions [12]. The skin lesions of NC/Nga mice are clinically and histologically very similar to human AD, and plasma levels of total IgE in conventional NC/Nga mice are markedly elevated from 8 weeks of age, correlating with clinical skin severity of dermatitis [12]. Therefore, NC/Nga mice are widely used as an animal model for human AD.

In the present study, we examined the effects of ASD exposure on the development of AD in NC/Nga mice.

## Materials and methods

### Animals

Specific pathogen-free male 12-week-old NC/Nga mice were purchased from Charles River Japan Laboratories (Atsugi, Kanagawa, Japan). The animals were housed in standard plastic cages, under conditions of controlled temperature (25°C), humidity, and lighting (lights on from 8:00 to 20:00). All animal experiments in this study were approved by the Animal Research Committee of Tsukuba University, and all animal work was conducted according to the University guidelines, and international guidelines.

### Analysis of sulfate, nitrate and elements, lipopolysaccharides, and β-glucan in particles

ASD used in the present study was collected from surface soils in the Shapotou Desert located on the southern fringe of the Tengger Desert in north central China [13], where dust storms occur frequently [14]. The particle diameter of the samples (a total of 600 particles) was measured using a scanning electron microscope (JSM-5800 JEOL Ltd., Tokyo, Japan). The size distribution peak was observed at 6 μm.

Sulfate (SO_4_^2−^) and nitrate (NO_3_^−^) in the samples were analyzed using an ion chromatograph (DX-100, Dionex, Sunnyvale, CA, USA). The concentration of each element was determined by inductively coupled plasma atomic emission spectroscopy (ICP-AES, 61E Trace and ICP-750, Thermo Jarrell-Ash, Grand Junction, MA, USA).

The contents of lipopolysaccharide (LPS) and β-glucan in each particle sample were measured by the kinetic assay using Endospec ES test MK (Seikagaku Cop., Tokyo, Japan) for LPS activity, and the Fungitec G test MK (Seikagaku Cop., Tokyo, Japan) for β-glucan activity, according to the manufacturer’s protocol. In brief, approximately 2.5 mg of each particle sample was suspended in 1 mL water (LPS and β-glucan free; Otuka Co., Kyoto, Japan) for 1 h, and was placed on the bench top at room temperature for 2 h. Next, the supernatants were recovered and tested for LPS and β-glucan concentration. The limits of detection in LPS and β-glucan assays were < 0.001 EU/mL and 2 pg/mL, respectively.

### Reagents and drugs

Heated Asian sand dust (H-ASD) was created by heating ASD at 360°C for 30 min under 80% nitrogen gas in an electric heater to exclude toxic materials (microbiological materials, sulfates, etc.)

Biostir-AD, an ointment-containing component of the house dust mite *Dermatophagoides farina* (Df), was purchased from Biostir Hyogo, Japan). Hydrophilic petrolatum (Maruishi pharmaceutical Co., Ltd, Osaka, Japan) was used as the control.

### Animal experiment protocol

AD-like skin lesions were induced in 12-week old male NC/Nga mice with Biostir-AD, according to the manufacturer’s protocol. The NC/Nga mice were divided into 6 groups: hydrophilic petrolatum only (control); hydrophilic petrolatum plus ASD (ASD); hydrophilic petrolatum and H-ASD (H-ASD); Biostir-AD (Df); Biostir-AD and ASD (Df + ASD), and; Biostir-AD and H-ASD (Df + H-ASD); n = 8 animals in each group. The hair on the upper back of the mice was shaved with clippers and a shaver. For barrier disruption, 150 μL of 4% sodium dodecyl sulfate (SDS; WAKO, Osaka, Japan) was applied to the shaved back skin, and both surfaces of each ear, 2 hours before application of the treatment. The amount of ASD or H-ASD used for the treatment was 150 μg each time, and that of Biostir-AD and hydrophilic petrolatum was 100 mg. In the groups of Df + ASD or Df + H-ASD, 150 μg of ASD or H-ASD was mixed with 100 mg of Biostir-AD, and in the ASD group, 150 μg of ASD was mixed with hydrophilic petrolatum. These procedures were repeated twice a week for 4 weeks.

### Dermatitis score

The severity of dermatitis was evaluated once a week, immediately prior to the treatment. The development of: 1) erythema/hemorrhage; 2) scarring/dryness; 3) edema, and; 4) excoriation/erosion, was scored as 0 (none), 1 (mild), 2 (moderate) and 3 (severe). The sum of the individual scores was taken as the dermatitis score [15]. Scratching behavior was measured every week with MicroAct (Neuroscience, Tokyo, Japan).

### Histopathological examination

After being humanly sacrificed, the back skins and ears of each mouse were fixed in 10% v/v formalin, and the tissues were embedded in paraffin and stained with Toluidine blue. Mast cell number was counted under a microscope at a magnification of × 200 at 5 sites chosen at random.

### Measurement of IgE concentration in serum

Blood was collected from each mouse at the end of the experiment. Total serum IgE levels were measured by enzyme-linked immunosorbent assay (ELISA) using the ELISA starter accessory kit (Bethyl Laboratories, Montgomery, TX, USA) and Mouse IgE antibody (Bethyl Laboratories) according to the manufacturer’s instructions. The absorbance was determined using a microplate reader.

### Statistical analysis

Results are expressed as means (±standard deviations, SD). Differences between groups were examined for statistical significance using Student’s *t* test. A P-value < 0.05 was considered significant.

## Results

The mean concentrations of SO_4_^2−^, LPS, and β-glucan in the ASD samples were 900 μg/g, 3.66 EU/mg, and 15.20 pg/mg respectively. NO_3_^−^ was present at a mean concentration of < 500 μg/g in the ASD group. However, none of the above-mentioned compounds were detected in the H-ASD samples. The contents of elements with oxide were 60% for SiO_2_; 11.1% for Al_2_O_3_; 4.1% for Fe_2_O_3_; 1.8% for Na_2_O; 9.0% for CaO; 2.5% for MgO; 0.7 for TiO_2_; 2.2% for K_2_O; whereas 8.6% were lost on ignition.

**Figure 1:**
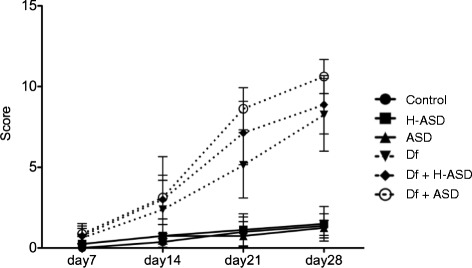
**Dermatitis scores using NC/Nga mice with various treatments.** Nc/Nga mice (n = 8 for each group) were treated with either hydrophilic petrolatum only (Control); hydrophilic petrolatum plus ASD (ASD); hydrophilic petrolatum and H-ASD (H-ASD); Biostir-AD (Df); Biostir-AD and ASD (Df + ASD), or; Biostir-AD and H-ASD (Df + H-ASD). The Y-axis represents the dermatitis score at each week.

Dermatitis scores using NC/Nga mice with the various treatments are shown in Figure 1. ASD alone did not induce AD-like skin symptoms. However, Df alone, Df + H-ASD and Df + ASD all induce AD-like symptoms, and the dermatitis score in the Df + ASD group was significantly higher compared to the Df group (P = 0.0011 at day 21, and P = 0.017 at day 28). No significance in the dermatitis score was observed between the Df group and the Df + H-ASD group (P > 0.05).

**Figure 2:**
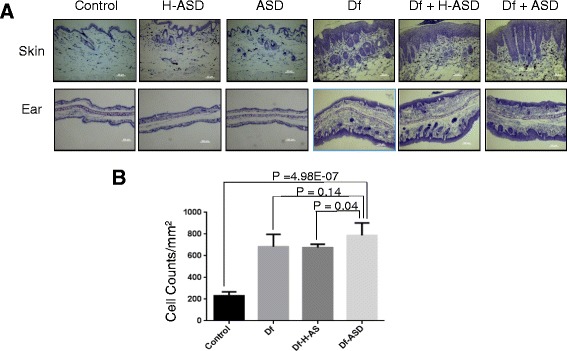
**Histological observation of NC/Nga mouse skin and ears by Toluidine blue staining. A)** Skin (upper row) and ear (lower row) of mice with the various treatments. No pathological changes were observed in the hydrophilic petrolatum treatment (control), H-ASD, and ASD group, but marked infiltration of the cells and increased epidermal thickness were observed in the Df, Df + H-ASD and Df + ASD treatment groups. **B)** Number of mast cells infiltrated in the skin after 4 weeks treatments.

When comparing the histological changes of the dorsal skin and ears on day 28 (Figure 2A), the lesional skin and ears showed a significant thickening of the dermis in the Df, Df + H-ASD and Df + ASD groups compared to the control group. Histopathological examination of the skin also revealed remarkable hyperkeratosis and marked acanthosis; severe inflammatory cell infiltration in the dermis was observed in the Df, Df + H-ASD and Df + ASD groups. In contrast, these histologic findings were not observed in the ASD, H-ASD, or control groups. Mast cell infiltration of the skin was markedly increased in the Df, Df + H-ASD and Df + ASD groups compared to the control group (P < 0.0001 for both, Figure 2B). The number of mast cells in the Df + ASD group tended to be higher compared to the Df group, but it did not reach statistical significance (P = 0.14, Figure 2B).

**Figure 3:**
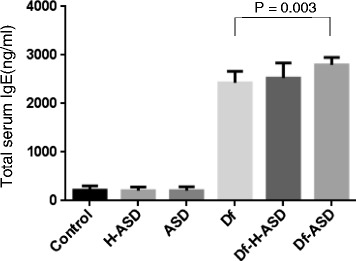
**Total serum IgE levels in Nc/Nga mice after the various treatments.**

Mean serum IgE levels were markedly increased in the Df, Df + H-ASD and Df + ASD groups, compared to the ASD, H-ASD and control groups (P < 0.0001 for both, Figure 3), and serum IgE levels in the Df + ASD group were significantly higher compared to the Df group (P = 0.003).

## Discussion

In the present study, we found that AD-like symptoms induced by a major allergen, Df, were enhanced by ASD in NC/Nga mice, while ASD alone did not cause AD-like symptoms. This is, in our knowledge, the first study reporting effects of ASD on the worsening of dermatitis symptoms in a murine model. ASD contain various materials, and a previous study reported that removal of toxic materials in ASD such as LPS and sulfates, caused very slight pathological changes in ovalbumin-induced allergic inflammation of mice [4]. Accordingly, our study showed that AD-like symptoms were enhanced by Df + H-ASD as well as Df + ASD, but that the degree of enhancement was greater in the Df + ASD group compared to the Df + H-ASD group. A recent study by He et al. compared two ASD samples that were collected from different regions, ASD1 from Inner Mongolia and ASD2 from northwest China [16]. The ASD samples contained different concentrations of LPS, β-glucan, and SiO_2_. LPS and β-glucan were present in higher concentrations in ASD2 than in ASD1, whereas the concentration of SiO_2_ was greater in ASD1 than in ASD2. The aggravating effects of ASD2 on lung eosinophilia were greater than those of ASD1, suggesting that the aggravation of lung eosinophilia may be dependent on LPS rather than on SiO_2_ in ASD.

Our data, along with that from previous studies, indicates that materials such as LPS in crude ASD may play a role in the exacerbation of allergic symptoms in combination with allergens.

It has been reported that ASD significantly increase gene transcription of interleukin (IL)-1, IL-6, IL-8, granulocyte-macrophage colony-stimulating factor (GM-CSF), caspase 14 (CASP14), and cytochrome P450 enzymes, including CYP1A1, CYP1A2, and CYP1B1, in human epidermal keratinocytes [17]. It has also been shown that ASD showed a dose-dependent increase in the protein release of IL-6 and IL-8 pro-inflammatory cytokines, on marrow-derived dendritic cells in mice, but these effects were not observed for H-ASD [18]. In our AD mice model, ASD alone did not cause AD-like symptoms in NC/Nga mice, but ASD, and not H-ASD, enhanced AD-like symptoms and allergic inflammation caused by the house dust mite, which is similar to the effects observed in mice challenged with ovalbumin combined with ASD and H-ASD in a previous study [19]. The same study reported that ASD contained various chemical and biological materials, and that these materials were mostly inactivated by heating at 360°C [19]. These data imply that the heat-inactivated materials in ASD may have an effect on the symptoms of the AD-like skin region in Nc/Nga mice.

A limitation of the study is that we did not observe a statistically significant difference in the mast cell numbers of the skin between the Df alone group and the Df + ASD group (P = 0.14, Figure 2B), but a statistically significant difference was observed between the Df + H-ASD group and the Df + ASD group (P = 0.04, Figure 2B). This may be attributable to the small sample size (n = 8 in each group), which was not sufficient to detect a statistically significant difference between the Df alone group and the Df + ASD group.

Epidemiological studies conducted in South Korea [20], Taiwan [21-23], and Japan, [24] have revealed that ASD storm events are associated with the worsening of respiratory symptoms, other health problems such as strokes, and increased mortality rates. It has also been demonstrated that airborne allergens such as pollens are absorbed onto ASD [17]. Although, to our knowledge, no epidemiological studies have been conducted for the association between ASD and the symptoms of dermatitis, our data suggest that it is likely that ASD may contribute to the exacerbation of not only respiratory symptoms, but also skin diseases, in susceptible individuals.
